# Implementation of the HiBalance training program for Parkinson’s disease in clinical settings: A feasibility study

**DOI:** 10.1002/brb3.1021

**Published:** 2018-06-21

**Authors:** Conran Joseph, Breiffni Leavy, Sara Mattsson, Lynn Falk, Erika Franzén

**Affiliations:** ^1^ Department of Neurobiology, Care Sciences and Society Division of Physiotherapy Karolinska Institutet Stockholm Sweden; ^2^ Faculty of Community and Health Sciences Physiotherapy Department University of the Western Cape Cape Town South Africa; ^3^ Unit of Research and Development Stockholms Sjukhem Foundation Stockholm Sweden; ^4^ Rehabilitation and Primary care Stockholms Sjukhem Foundation Stockholm Sweden; ^5^ Allied Health Professionals Function Karolinska University Hospital Stockholm Sweden

**Keywords:** balance training, feasibility, implementation, Parkinson’s disease

## Abstract

**Background:**

Translating evidence into practice requires adaptation to facilitate the implementation of efficacious interventions. A novel highly challenging balance training program (HiBalance) was found to improve gait, balance, and physical activity in persons with Parkinson’s disease (PD) in an earlier randomized controlled trial. This study aimed to describe the adaptation process and feasibility of implementing the HiBalance program for PD within primary healthcare settings.

**Method:**

Feasibility was assessed in terms of study processes and scientific evaluation. Nine persons with mild–moderate PD were enrolled in this pre–post feasibility study. The dose of the original program was adapted by reducing therapist‐led training sessions from three to two times weekly. Outcome measures were substituted with ones more clinically feasible. One group (*n* = 5) received HiBalance training three times weekly for 10 weeks while another (*n* = 4) trained twice weekly plus a once weekly home exercise program (HEP). Balance performance was the primary outcome, while secondary outcomes (e.g., gait speed, physical activity level, concerns of falling, and health‐related quality of life) were also evaluated.

**Results:**

Regarding process feasibility, attendance was high (approximately 90%) in both groups, and experiences of the group and home training were positive. Newly selected outcome measures were feasible. The scientific evaluation revealed few adverse events and no serious injuries occurred. Concerning outcomes per group, the average change in balance performance and gait speed was equal to, or exceeded, the minimally worthwhile treatment effect commonly used in PD.

**Conclusion:**

The findings support the feasibility, in terms of process and scientific evaluation, of the adapted HiBalance program for implementation within clinical settings. A sufficiently powered study is required to ascertain whether the newly proposed program offers similar short and long‐term effects as the original program.

## 1 INTRODUCTION

The implementation of efficacious interventions into healthcare settings is necessary for improving the health of larger patient groups (Bradley et al., 2004). However, the rate at which promising interventions are embedded within healthcare remains suboptimal because of challenges related to implementation (Proctor et al., 2011). The initial demands and costs to society are considerable for implementing new evidence (Krisberg, 2010) therefore, investigating feasibility aspects (e.g., process and scientific evaluation) is important for ensuring optimal uptake of interventions.

Parkinson’s disease (PD) is a neurodegenerative disorder typically resulting in deterioration of gait and balance abilities which predispose individuals to more frequent falls and injuries (Bloem, Grimbergen, Cramer, Willemsen, & Zwinderman, 2001). To combat‐associated symptoms, the HiBalance training program has been designed, including highly challenging and progressive exercises targeting dysfunctions of subsystems of balance control among persons with mild‐to‐moderate PD (Conradsson, Lofgren, Stahle, Hagstromer, & Franzen, 2012). To date, the intervention has been found to be feasible (Conradsson, Lofgren, Stahle, & Franzen, 2014) and effective at improving balance and gait performance, activities of daily living, and physical activity levels in more controlled settings, that is, randomized controlled trials (Conradsson et al., 2015). In order to reach a larger proportion of people with PD, the next step involves testing the clinical applicability and implementation of the program.

Translating research protocols into clinical practice is not a straightforward task because a complex set of decisions and compromises needs to be made in order to be considered by healthcare planners and implementers. However, protocol changes are rarely examined in the literature, especially related to rehabilitation interventions which are inherently complex. The objective of this study was to describe the adaptation process, specifically the necessary changes needed to translate a research protocol into clinical practice, as well as procedural and scientific feasibility of the adapted HiBalance program for implementation within primary healthcare settings.

## 2 MATERIALS AND METHODS

### 2.1 Design

This feasibility study had been approved by the regional board of ethics in Stockholm. A typology for feasibility studies, according to Thabane et al., (2010) was used to gather insight into aspects related to translating an intervention from more controlled to clinical settings. This typology provides useful information prior to conducting large‐scale studies by providing insight into the following assessments: (1) processes, (2) resources, (3) management, and (4) scientific (effectiveness). For the purpose of this study, the specific *process* and *scientific* aspects of feasibility, as summarized in Table 1, were investigated.

**Table 1:** Primary purposes of feasibility studies and those targeted in the current study

Main reason for conducting pilot/feasibility studies	Aspects commonly assessed
Process^a^This assesses the feasibility of the processes that are key to the success of the main study	Recruitment and retention rates (Non)compliance or attendance rates Eligibility criteria ~ sufficient or restrictive Appropriateness and understanding of data collection tools/outcome measures Length of time to complete all study forms
ResourcesThis deals with assessing time and resource problems that can occur during the main study	Determining center willingness and capacity Determining process time Is the equipment readily available when and where needed?
ManagementThis covers potential human and data management problems	What are the challenges that participating centers have with managing the study? What challenges do study personnel have?
Scientific^a^This deals with the assessment of treatment safety, dose, response, effect, and variance of the effect	Is the intervention safe? What is an effective dose level? Do patients respond to the intervention? What is the estimate of the treatment effect?

*Note*

a Indicates feasibility aspects investigated in this study.

### 2.2 Participants

Participants were consecutively recruited from a convenience sample of referrals at one primary care rehabilitation clinic in central Stockholm. Fourteen participants were invited for initial screening, where nine met the following inclusion criteria: (1) a diagnosed of idiopathic Parkinson’s disease; (2) mild–moderate disease severity according to a Hoehn & Yahr, (1967) score of 2 or 3; (3) absence of noteworthy cognitive impairment; (4) age ≥60 years; (5) ability to independently ambulate indoors without the use of a mobility aid; and (6) being on a stable dose of anti‐Parkinson’s medication for ≥3 weeks. Five participants did not meet the preliminary inclusion criteria: one due to nonidiopathic PD; two had severe impairments (cognitive and balance); and two with comorbidities (extreme back and hip pain) which could have impacted study outcomes. Written informed consent was obtained before first assessments were carried out. As per Table 2, the average age was 71 years and subjects were predominantly female (6/9). The average time since diagnosis was 11 years, and one‐third experienced a fall during the past 12 months.

**Table 2:** Participants’ baseline characteristics

Subjects	Gender	Age (years)	H&Y stage	Mini‐BESTest	Gait speed (m/s)
Group 3x					
P1	F	68	2	24	1.26
P2	M	75	2	23	1.29
P3	F	83	3	16	0.79
P4	F	70	3	19	1.03
P5	F	61	2	27	1.36
Group 2x + HEP					
P1	M	69	3	21	1.14
P2	F	76	2	21	1.14
P3	M	70	2	24	1.12
P4	F	66	2	23	0.91

*Note*

Group 3x: received supervised balance training three times weekly for 10 weeks. Group 2x +HEP: received supervised balance training twice weekly for 10 weeks plus a once weekly HEP.

Due to the feasibility design, with our sole interest in assessing the translation of the HiBalance training program from theory (RCT environment) to practice (clinical settings), no sample size calculation was needed and performed. Results from the process and scientific feasibility will be used to inform the larger implementation trial.

### 2.3 Adaptation of the HiBalance program

The theoretical underpinnings of the *HiBalance* program have previously been described in detail (Conradsson et al., 2012, 2014; Leavy, Kwak, Hagströmer, & Franzén, 2017). This program targets subsystems of balance control typically affected in PD: (1) sensory integration, (2) anticipatory postural adjustments, (3) motor agility, and (4) stability limits. To target these progressive symptoms, motor learning principles, that is, specificity, progressive overload, and variation, were used as foundation to challenge individual progression. The program is group‐based (4–7 persons) and is facilitated by two physical therapists (PT’s) who were trained to develop exercises according to the balance control framework used in this study. Additionally, the program incorporates gradual integration of dual‐tasking (DT)—cognitive (e.g., counting or remembering items) and motor task (e.g., carrying or manipulating an object)—to target mild‐associated cognitive impairments.

Adaptation of certain aspects of the initial balance training program was necessary to facilitate its implementation within clinical settings. Firstly, the three times weekly training dose during the efficacy trial was reduced to align with policy regarding rehabilitation reimbursement in the Swedish healthcare system. Training dosage was discussed during a workshop by an expert group consisting of PT’s from hospitals and primary care facilities, including some with previous experience of the intervention, and the researcher group. The final decision was to reduce therapist‐led sessions to twice weekly for 10 weeks. This decision was also informed by data from a previous qualitative study where participants perceived that 30 training sessions were too great a time commitment (Leavy, Roaldsen, Nylund, Hagstromer, & Franzen, 2016).

To compensate for this marked reduction, a home exercise program (HEP) was proposed and developed over several months by the same expert group during a workshop and thereafter circulated for comments, where progression and variation aspects of exercises were added. Patients attending rehabilitation clinics in Sweden are always given home exercise programs on top of their rehabilitation interventions to aid self‐management and promote overall cardiovascular fitness. This adjunct program mainly focuses on aerobic capacity, strengthening of lower extremity and core muscles—components that can be performed unsupervised with minimal risk of falls, and which have been shown to improve balance control and gait in PD (Roeder, Costello, Smith, Stewart, & Kerr, 2015; Kahle & Tevald, 2014). The final program included 20 therapist‐led and 10 individual home exercise sessions. Although the core components of the program were left unchanged, it was necessary to determine the feasibility of the newly proposed training schedule by investigating whether the intended effects were still achievable.

The other aspect for adaptation regarded the selection of outcome measurement. Clinically applicable outcome measures were required to replace laboratory‐based measures used during the efficacy trial. Additionally, time efficient measurements were needed to ensure clinical feasibility. Consequently, the test battery was condensed to fewer performance‐based and self‐reported measures (Leavy et al., 2017). The primary outcome, that is, balance performance measured with the Mini‐BESTest, remained unchanged.

### 2.4 Feasibility of the adapted HiBalance program

#### 2.4.1 Process assessment

For the assessment of process feasibility, attendance/adherence rates, eligibility criteria, the appropriateness of data collection tools/outcome measures, time taken to complete all measurements, and participants’ experiences of the program were investigated. *Attendance rate* was measured by recording individual participation over the 10‐week period and reasons for missed sessions. The *eligibility criteria* reflected the efficacy trial: persons ≥60 years of age with mild–moderate PD. Clinically applicable outcome measures were selected by experts at the workshop to replace laboratory‐based tests. The Mini‐BESTest, (14‐item performance‐based measure of dynamic balance), was used as in the efficacy trial. Conversely, gait speed was manually assessed with the timed 10‐m walk test, as opposed to the electronic walkway. The 6‐min walk test was added to evaluate exercise endurance. Lastly, accelerometery (Actigraph GT3X +, Pensacola, USA) measured free‐living physical activity by aggregating step counts. Self‐reported measures included the Falls Efficacy Scale–International (FES‐I) for evaluating concerns of falling, the Walk‐12 (Swedish version Walk‐12G) evaluated self‐perceived limitations in walking ability, and the EQ‐5D assessed health‐related quality of life. Time taken to complete testing was recorded for all individuals. Upon program completion, participants reported their perception of the balance training and HEP using a questionnaire.

#### 2.4.2 Scientific assessment

Safety of the intervention was assessed by recording the nature and frequency of adverse events which included falls, injuries, fatigue, and pain. To assess the effectiveness (responsiveness) of the newly proposed dose, participants were randomized to (1) receive training three times weekly (Group 3x), (2) receive group training twice weekly and perform the HEP once a week (Group 2x + HEP).

### 2.5 Data analysis

Data were analyzed descriptively, using the proportion of individuals per group who changed their outcome status at post‐testing. This study was not sufficiently powered to test between‐group differences and report on the statistical superiority of one training dose over the other. However, we discussed the observed changes in relation to their clinically meaningful important differences.

## 3 RESULTS

### 3.1 Process assessment: attendance, data collection procedure, and experiences

Group 3x attended 137 of 150 sessions (91%) and Group 2x + HEP, 74 of 80 therapist‐led sessions (93%) and 32 of 40 (80%) home training sessions. All data collection procedures, that is, self‐reported outcomes and clinical tests, were completed with ease by participants, indicating the clinical feasibility of selected measures. Measurements took between 90 and 120 min to complete. Concerning recruitment, a number of patients under 60 years, but who fulfill all other criteria, was excluded, which suggested broadening of the inclusion criteria. Both groups reported therapist‐led sessions as balance challenging and exercise difficulty as progressive in nature, especially with the introduction of dual‐tasking. All participants, including the subject who had missed some official HEP sessions, in the Group 2x + HEP, expressed their willingness to continue with the HEP after the intervention period.

### 3.2 Scientific assessment: safety and effectiveness

Concerning adverse events during therapist‐led sessions, three participants fell without causing injury; all stumbled over artificial hindrances that were created to challenge balance. Another participant reported pain associated with the training which lasted more than 2 days, and another felt dizzy during the session but recovered after a resting period. No participant reported negative events during the HEP.

**Figure 1:**
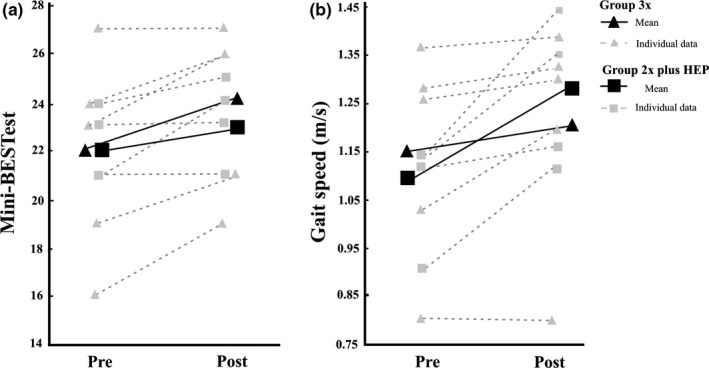
(a) Mini‐BESTest scores and (b) gait speed pre‐ and postintervention

Four of five individuals in Group 3x improved their balance performance and two of four in Group 2x + HEP, while no one reduced their balance function (Figure 1). The average improvement in balance performance following the training period was two points and one point in Group 3x and Group 2x + HEP, respectively. Four of five (80%) in Group 3x, and all (100%) in Group 2x + HEP, improved their gait speed following training. Those in Group 3x improved their gait speed by 0.05 m/s on average, whereas those in Group 2x + HEP improved it by 0.17 m/s. Furthermore, both groups improved their walking endurance (6MWT) and four of five individuals in Group 3x, and three of four in Group 2x + HEP, indicated fewer concerns of falling following training. No noteworthy differences in pre–post measures were found for physical activity level, self‐perceived walking ability, and health‐related quality of life (EQ‐5D) after the intervention.

## 4 DISCUSSION

Results of this study reveal that the adapted HiBalance program was feasible in mild–moderate PD within a primary healthcare setting, in that attendance rates were high, adverse events few, and the effects on balance performance and secondary outcomes were detectable and favorable. The results support initial feasibility of the intervention which was adapted to suit the healthcare context. However, a larger and more rigorously designed multisite study is required to establish whether the newly adapted program offers similar short‐ and long‐term effects as the original program.

According to the feasibility typology, assessment of the process revealed good adherence as approximately 90% of the total sessions were attended by participants. The eligibility criteria were similar to that used in the RCT (Conradsson et al., 2015) however, the recruitment process revealed that younger patients (<60 years) are commonly referred to rehabilitation in primary healthcare settings. To promote access, the implementation trial will include persons of all ages. Participants completed the newly selected outcome measures with ease. This could be because some of the selected measures, such as the EQ‐5D and 6MWT, are part of existing routine practice. However, PT’s reported that almost 2 hr of data collection per participant was not clinically feasible, and the 6MWT was resultantly removed from the test battery, while the Walk‐12, FES‐I, and EQ‐5D were suggested to be completed by participants at home. Overall, participants’ experiences were positive and all felt challenged by the program.

As part of the scientific evaluation, adverse events were few during the therapist‐led training program and HEP. Three falls were recorded during the training sessions, with all of them occurring during the advance stages of exercise progression. All falls occurred on a soft surface and no injuries were reported. Risk of falls, however, cannot be removed during training sessions due to the highly challenging nature of the program. To minimize falls, challenging balance exercises were not included in the HEP, while PT’s remained near participants during training sessions.

Training resulted in improved balance performance. Despite the small sample size, both groups improved their balance performance by at least one point on the Mini‐BESTest, which is also the minimal detectable change in mild–moderate PD (Löfgren, Lenholm, Conradsson, Ståhle, & Franzén, 2014). Similar positive trends were found for secondary outcomes, that is, gait speed, concerns of falling, and exercise endurance. In fact, a similar (average) difference in gait speed was found following training in the Group 2x + HEP when compared to the efficacy trial (Conradsson et al., 2015). The fact that the findings of the two studies corroborate are an indication of the potential effectiveness of the adapted training dose in the current study.

This study presented with several limitations. Firstly, the sample size was too small to sufficiently power outcomes and to adequately evaluate adverse events. Also, the lack of a control group undermined true treatment effects. Lastly, the lack of assessor blinding may have introduced measurement bias. Taken together, future larger and more rigorously designed studies are warranted to provide evidence on the cost‐effectiveness of the adapted HiBalance training program within clinical settings. Translating this training program into practice may offer clinicians an effective option to retrain and maintain balance and gait in persons with PD, an area that lacked proven interventions (Conradsson, Leavy, Hagströmer, Nilsson, & Franzén, 2017; Tomlinson et al., 2012).

In conclusion, this study demonstrates that the adapted HiBalance program is feasible from both a process and scientific/effectiveness perspective. A larger multisite study is needed to test the effectiveness of the adapted program on balance and gait outcomes as well as to inform future widespread implementation.

## CONFLICT OF INTEREST

All authors have no conflict of interest to declare.
